# Exosomal FZD-7 Expression Is Modulated by Different Lifestyle Interventions in Patients with NAFLD

**DOI:** 10.3390/nu14061133

**Published:** 2022-03-08

**Authors:** Maria Principia Scavo, Nicoletta Depalo, Federica Rizzi, Livianna Carrieri, Grazia Serino, Isabella Franco, Caterina Bonfiglio, Pasqua Letizia Pesole, Raffaele Cozzolongo, Vito Gianuzzi, Maria Lucia Curri, Alberto Ruben Osella, Gianluigi Giannelli

**Affiliations:** 1 Laboratory of Personalized Medicine, National Institute of Gastroenterology “S. De Bellis” Research Hospital, Via Turi 27, Castellana Grotte, 70013 Bari, Italy; livianna.carrieri@irccsdebellis.it; 2 Institute for Chemical-Physical Processes, Italian National Research Council (IPCF)-CNR SS Bari, Via Orabona 4, 70125 Bari, Italy; federica.rizzi@uniba.it (F.R.); marialucia.curri@uniba.it (M.L.C.); 3 Dipartimento di Chimica, Università degli Studi di Bari Aldo Moro, Via Orabona 4, 70125 Bari, Italy; 4 Molecular Medicine, National Institute of Gastroenterology “S. de Bellis”, Research Hospital, Via Turi 27, Castellana Grotte, 70013 Bari, Italy; grazia.serino@irccsdebellis.it; 5 Laboratory of Epidemiology and Biostatistics, National Institute of Gastroenterology “S. De Bellis” Research Hospital, Via Turi 27, Castellana Grotte, 70013 Bari, Italy; isabella.franco@irccsdebellis.it (I.F.); catia.bonfiglio@irccsdebellis.it (C.B.); arosella@irccsdebellis.it (A.R.O.); 6 Department of Pathology, National Institute of Gastroenterology “S. de Bellis”, Research Hospital, Via Turi 27, Castellana Grotte, 70013 Bari, Italy; letizia.pesole@irccsdebellis.it; 7 Department of Gastroenterology, National Institute of Gastroenterology, “S. de Bellis” Research Hospital, Castellana Grotte, Via Turi 27, Castellana Grotte, 70013 Bari, Italy; raffaele.cozzolongo@irccsdebellis.it (R.C.); vito.giannuzzi@irccsdebellis.it (V.G.); 8 Scientific Direction, National Institute of Gastroenterology “S. de Bellis”, Research Hospital, Castellana Grotte, 70013 Bari, Italy; gianluigi.giannelli@irccsdebellis.it

**Keywords:** NAFLD, plasma-derived exosomes, Frizzled 7, low glycemic index Mediterranean diet, physical activity

## Abstract

Non-alcoholic fatty liver disease (NAFLD) is a multifactorial condition characterized from hypertriglyceridemia and hepatic fat accumulation, in the absence of alcohol intake. NAFLD starts as steatosis (NAFL), and the continued injury relative to the toxic fat induces inflammation, steatohepatitis (NASH), and HCC. One of the factors determining liver degeneration during the evolution of NAFLD is a modification of Wnt/Frizzled (FZD) signaling. In particular, an inhibition of Wnt signaling and an overexpression of a specific FZD receptor protein, namely, the FZD7, have been observed in NAFLD. Actually, the prognosis and the follow-up of NAFLD is not easy, and the liver biopsy is the gold standard for an accurate detection of liver fibrosis. In this study, the modulation of the FZD7 expression levels in plasma-derived exosomes of NAFLD-affected patients, before and after specific lifestyle interventions, were experimentally evaluated by Western blotting analysis. The experimental data were analyzed by an accurate statistical study that indicated, in the exosomes derived from plasma of NAFLD patients with moderate or severe steatosis, an average expression level of FZD7 that was significantly higher than healthy subjects at baseline; conversely, the values were normalized after 90 days of specific lifestyle interventions. The overall results suggested that the FZD7 delivered by exosomes represents a good candidate as a new and effective biomarker for diagnosis and prognosis of NAFLD.

## 1. Introduction

Non-alcoholic fatty liver disease (NAFLD), a multifactorial complex chronic condition, is considered a metabolic syndrome with hypertriglyceridemia and abnormal liver fat accumulation, without alcohol intake or use of steatogenic drugs. The relevant contributions to the epidemiology of NAFLD has been attributed to overweight and obesity that represent the condition, resulting from sedentary lifestyle combined with unregulated diet. NAFLD not only covers a wide spectrum of liver or hepatic diseases, but also induces extra-hepatic consequences including cardiovascular diseases and diabetes [1,2,3]. NAFLD starts as simple steatosis (NAFL), abnormal accumulation of toxic liver fat triggers inflammation, inducing the development of the so-called non-alcoholic steatohepatitis (NASH). Therefore, NASH, a chronic state of liver inflammation, causes the transformation of hepatic stellate cells to myofibroblasts with the concomitant production of extra-cellular matrix (ECM), thus progressing in liver fibrosis. Furthermore, advanced liver fibrosis represents a relevant risk factor for development of hepatocellular carcinoma (HCC), that HCC has also been found also in patients with NAFLD in the absence of cirrhosis [4].

It well known that inflammation process in NASH progression is due to different factors, including lipotoxicity, immune response, and gut microbial dysbiosis. It is also well documented that aberrations in the Wnt signaling can lead not only to cancer but also to liver disease [5]. Similarly to what happens for other organs, the Wnt/β-catenin signaling pathway is fundamental in liver development and biology (liver regeneration and basically for normal liver condition of metabolism). Its regulation is determined by the Wnt/Frizzled (FZD) receptor interaction; during prenatal liver development, all the 10 FZD receptor genes have been detected [6,7]. FZD proteins, which are G protein-coupled receptors (GPCRs), play an important role during cancer development; indeed, they are described as cancer drivers. The dysregulation of FZD receptors has been found to occur in HCC, and the active functional role of FZD receptors in liver deterioration and HCC progression has been proven [8]. The expression profile of Wnt signaling in the NAFLD has suggested an inhibition of Wnt signaling, with a consequent increase of expression of a specific FZD receptor protein, namely, the FZD7 [9]; in effect, this protein has been found correlated to the grade of steatosis and degeneration of hepatic parenchyma. In particular, a study has revealed that upregulation of FZD7 is correlated with increased expression of wild-type β-catenin in HCC, thus suggesting that the Wnt/β-catenin signaling pathway in HCC can be activated through the FZD7 [7]. Experimental evidence has also suggested an involvement of the specific FZD proteins delivered by exosomes in the processes of tissue degeneration from normal to pathological in gastro-enteric diseases, especially in metastasis spread, inflammation, and in the evolution of metabolic disease [10]. Exosomes, which are well recognized active cell–cell communicators, produced from all cells present in parenchymal and non-parenchymal departments, have been proved able to deliver their bioactive cargo from cell of origin to recipient cells. As reported from Sasaki and colleagues, the exosomes play a relevant role in many liver injuries, such as alcoholic and non-alcoholic fatty liver disease, liver fibrosis, cirrhosis, and HCC, as they are carriers of proteins, microRNA (miRNA), long noncoding RNA (lncRNA), and messenger RNA (mRNA) [11]. Exosomes, which are well recognized active cell–cell communicators, are produced not only from parenchymal cells but also from non-parenchymal cells; it has been proven that they are able to deliver their bioactive cargo from cell of origin to recipient cells. In the literature, their relevant involvement in NAFLD disease progression and degeneration has been extensively documented, as they promote inflammation [12,13,14,15].

Currently, prognosis in patients with NAFLD is not easy to make. Unfortunately, NAFLD is largely asymptomatic and, clinically, liver biopsy still represents the most reliable procedure for accurate detection of liver fibrosis. Indeed, determination of the stage of liver fibrosis allows for the prediction of the NAFLD progression towards liver-related mortality or other comorbidities. Consequently, development of non-invasive diagnostic tools is needed for NAFLD prognosis. In this perspective, deep understanding of the pathogenesis of NAFLD is crucial for identification of novel and reliable biomarkers for its diagnosis and prognosis that actually result poor or only hypothetical.

In the light of this evidence, the aim of this study was to evaluate the modulation of the FZD7 expression levels in plasma-derived exosomes of NAFLD-affected patients, before and after specific lifestyle interventions, as well as to estimate the effectiveness of the specific interventions on downregulation of the protein, in order to investigate on the potential use of the FZD7 as novel diagnostic and prognostic biomarker of NAFLD.

## 2. Materials and Methods

### 2.1. Patients’ Diagnostics Assessment

Quantification of fat content in the liver for NAFLD diagnosis in patients was performed by evaluating the ultrasound attenuation coefficient that can measured by the not invasive controlled attenuation parameter (CAP) technique at the standardized frequency of 3.5 MHz, taking advantage of a technology named vibration-controlled elastography (VCTE™), with a sensitivity higher than 85% [15,16] and implemented on Fi-broScan^®^ (Echosens, Paris, France) [17]. NAFLD was categorized as absent (<215 dB), mild (215–250 dB), moderate (251–299 dB), and severe (≥300 dB) [18,19]

### 2.2. Patient Enrollment

The study is part of the NUTRIATT (NUTRItion and AcTiviTy) study, a randomized clinical trial on the effect of diet and physical activity on NAFLD. Details concerning the patient recruitment have been published previously [20,21].

This is an overview of 166 subjects referred by the General Practitioners to the Laboratory of Epidemiology and Biostatistics of the National Institute of Digestive Diseases, IRCCS “S. de Bellis”, Castellana Grotte, Bari, Italy, or identified through NutriEp enrollment or follow-up [22,23]. Patients were enrolled, and the trial was registered at https://clinicaltrials.gov accessed on 31 December 2020 (registration number CT02347696). All subjects signed a consent permission form, and the study was conducted in accordance with the Declaration of Helsinki and approved by the Ethical Committee (Prot. n. 10/CE/De Bellis, 3 February 2015). Originally, the study was a parallel group randomized controlled clinical trial. The inclusion criteria concerned the following: (1) body mass index (BMI) ≥ 25, expressed as weight in kilograms divided by square height in meters (kg/m^2^); (2) age > 30 years and <60 years old; and (3) moderate or severe NAFLD as assessed by the controlled attenuation parameter (CAP). Exclusion criteria included: (1) overt cardiovascular disease and revascularization procedures; (2) stroke; (3) clinical peripheral artery disease; (4) T2DM (current treatment with insulin or oral hypoglycemic drugs, fasting glucose >126 mg/dL, or casual glucose >200 mg/dL); (5) alcohol intake higher than 20 g/day; (6) any severe medical condition that could prevent the subject’s participation in a nutritional intervention study; (7) subjects following a special diet, involved in a program for weight loss, or having experienced recent weight loss; and (8) inability to follow a low-glycemic index Mediterranean diet (LGIMD) for religious or other reasons. During the study, trained nutritionists interviewed patients with regard to socio-demographic factors, medical history, and lifestyle. Blood samples, collected by venous puncture in the morning after overnight fasting, were collected in tubes containing ethylenediamine tetra acetic acid (K-EDTA) anticoagulant. Biochemical measurements were performed using standard methods. All measurements were performed at baseline (T0) and at day 90th (T2). The subjects also underwent a fitness test to evaluate the initial physical condition, to identify the right training program, and finally to be able to compare the initial with the FU assessment of physical condition [24,25].

### 2.3. Interventions

The participants were randomly assigned to one of six intervention arms: (1) a control diet named the Inram diet (CD); (2) low-glycemic index Mediterranean diet (LGIMD); (3) physical activity 1 (PA1); (4) physical activity 2 (PA2); (5) LGIMD + PA1; and (6) LGIMD + PA2. The CD was based on CREA-AN guidelines [26], while the LGIMD was based on the work of Misciagna et al. [27]. No indication was given regarding the total calories to be consumed. Foods in LGIMD all have a low glycemic index (GI) and less than 10% total daily calories coming from saturated fats. The LGIMD had a high content in monounsaturated fatty acids (MUFA) from olive oil and also contained omega-3 polyunsaturated fatty acids (3PUFA) from both plant and marine sources. The prescribed diets were provided in a brochure format (see Supplementary Information), with graphic explanations. All participants were asked to record what they ate on a daily diary.

Physical activity interventions included two different types of exercise programs. The first PA1 included three non-consecutive sessions per week of moderate intensity aerobic activity (60–75% max HR, 3.0–5.9 METs distributed as follows: week 1–4: 14.2 kcal × kg^−1^ × week^−1^; week 5–8: 18.9 kcal × kg^−1^ × week^−1^; week 9–12: 23.6 kcal × kg^−1^ × week^−1^). The approximate duration of each session was between 50 and 60 min. Aerobic exercise intensity was monitored every 5 min using an automated heart rate monitor. Total weekly exercise duration was 150/180 min. Exercise modalities included treadmill walking, cycling, cross-training, and rowing, according to the aerobic activity program.

The second program, PA2, was based on the combination of aerobic activity and resistance training and included three non-consecutive sessions of 45 min of moderate intensity aerobic exercise on treadmill, cycling, rowing, and cross-trainer (60–75% max HR, 3.0–5.9 METs distributed as follows: week 1–4: 14.2 kcal × kg^−1^ × week^−1^; week 5–8: 18.9 kcal × kg^−1^ × week^−1^; week 9–12: 23.6 kcal × kg^−1^ × week^−1^); (2) 3 sets of 12 exercises, each to volitional fatigue such as leg press, adductor/abductor machine, gluteus machine, bicep curls, triceps extension, three different abdominal exercises, leg machine, low row, shoulder flexion. The weightlifting was increased by 1–2.5 kg × week^−1^ when 10 repetitions were completed in good form. The total weekly exercise duration was 180/240 min. Subjects were required to participate in a physical training program in a local gym, and they were able to decide the schedule for training and interact with the trainer at any time.

In this study, data from 95 randomly selected NAFLD subjects (56 males and 39 females, 38 with moderate steatosis and 57 with severe steatosis) who participated in the original trial and 20 subjects without NAFLD (10 males and 10 females) coming from a population-based random selected cohort assembled in this area of Southern Italy between 2005 and 2007 were considered; overall, 115 participants were involved. All healthy subjects were followed up between 2016 and 2018, using the same criteria applied to the NAFLD patients.

### 2.4. Exosome Isolation

Plasma specimens from all the enrolled subjects were processed for the exosome extraction by following the protocols reported in the literature [28,29]. Briefly, venous sampled blood specimens collected from all the subjects were kept at room temperature for 30 min, and then centrifuged at 4 °C for 10 min at 1500× *g* in ethylenediaminetetraacetic acid (EDTA). The plasmas were transferred to a clean tube and centrifuged again at 1.800× *g* for 10 min at 4 °C; then, the supernatants were transferred into screwcap cryovial. Typically, 5 mL of whole blood yielded about 1.5 mL of plasma that was then divided into aliquots of 500 µL and frozen at −80 °C when not immediately processed. The plasma aliquots were centrifuged at 3000× *g* for 15 min at 4 °C, and after transferring the supernatants into a clean tube, they were centrifuged at 3800× *g* for 15 min at 4 °C. Afterwards, the plasma specimens were ultra-centrifuged (BECKMAN, L-60 Ultracentrifuge) at 75,000× *g* for 1 h at 4 °C. Finally, the resulting supernatants were transferred into another clean ultracentrifuge tube, and a second ultracentrifugation cycle was performed at 100,000× *g* for 1 h and 30 min. Exosomes were collected as pellet and diluted in 200 µL of ultrapure water. For each sample, 50 µL of exosome suspension was immediately processed for DLS (dynamic light scattering) and TEM (transmission electron microscopy) analysis, while the left sample was stored at −80 °C until the protein extraction was carried out.

### 2.5. Dynamic Light Scattering and ζ-Potential Investigation

The exosome characterization in terms of size distribution, average hydrodynamic diameter, and colloidal stability was performed by using a Zetasizer Nano ZS, Malvern Instruments Ltd., Worcestershire, UK (DTS 5.00), as previously reported [22,23,24,25,26,27,28,29,30,31].

### 2.6. Transmission Electronic Microscopy Investigation

Exosomes were characterized by TEM using a Jeol JEM-1011 microscope at an accelerating voltage of 100 kV. TEM images were acquired using an Olympus Quemesa camera (11 Mpx) [30]. Staining of exosome samples, after their casting on TEM grids, was performed as reported by Latronico et al. [31].

### 2.7. Protein Extraction and Quantification from Exosomes

The exosome samples obtained from all subjects enrolled in the study were homogenized using 1 × radio immunoprecipitation buffer (RIPA, Cell Signaling Technology, Danvers, MA, USA) containing protease inhibitor (Amresco, Solon, OH, USA). For each exosomes sample, a Bradford kit assay (Bio-Rad Hercules, CA, USA) was used to quantify the total protein content. Anti-FZD7 (1:200 abCam Cambridge) and anti-ALIX (1:200 Cell signaling) were used to perform the immunoblotting assay, according to a previously reported procedure [32]. The chemiluminescence signals from proteins were imaged on the blotting membranes by using an enhanced chemiluminescence kit (Bio-Rad, Hercules, CA, USA) using ChemiDoc XRS+ (Bio-Rad, Hercules, CA, USA). Image Lab 5.2.1 software was used to analyze the images.

Furthermore, the ELISA test was performed to quantify the FZD7 expression level in the exosomes isolated from the plasma of 15 patients with moderate grade of steatosis, and of 15 patients with a severe grade of steatosis, at the enrollment time (T0) and after 90 days (T2), and of 10 control subjects. For this purpose, the quantitative Human Frizzled Homolog 7 (FZD7) ELISA Kit (Mybiosurse.com) was used, according to the protocol indicated by the manufacturer. The optical density (O. D.) was read at 450 nm within 10 min after adding the stop solution by using a Bio-RAD spectrophotometer.

### 2.8. Statistical Analysis

Data description was performed by means (SD), median (IQR), and frequencies (%) as appropriate. ANOVA was performed to test differences in the mean FZD7 expression between control and intervention subjects, NAFLD severity, and time (baseline vs. 90th day) and intervention arm. Age was included as a continuous variable in all analyses. As there were no differences in FZD7 mean expression levels between male and female subjects, sex was not included in the analyses. Post-estimation commands, such as margins and contrast, were used to describe the results and graphically display them. In particular, marginal estimations were obtained to probe the modification effect of NAFLD severity and time and to test the contrast between mean FZD7 expression as compared with a reference category. In all analyses, control subjects at baseline were the reference category. Statistical significance was set to *p* ≤ 0.05. Furthermore, post-estimation analysis by using Bonferroni test for multiple comparisons showed statistical significative decreased values for CAP values, which are reported in the Supplementary Information (Table S1). Stata (version 16.1) statistical package was used to perform all statistical analyses (StataCorp, 4905 Lakeway Drive, College Station, TX, USA).

## 3. Results

### 3.1. Evaluation of the FZD7 Expression Levels in Plasma-Derived Exosomes

**Figure 1:**
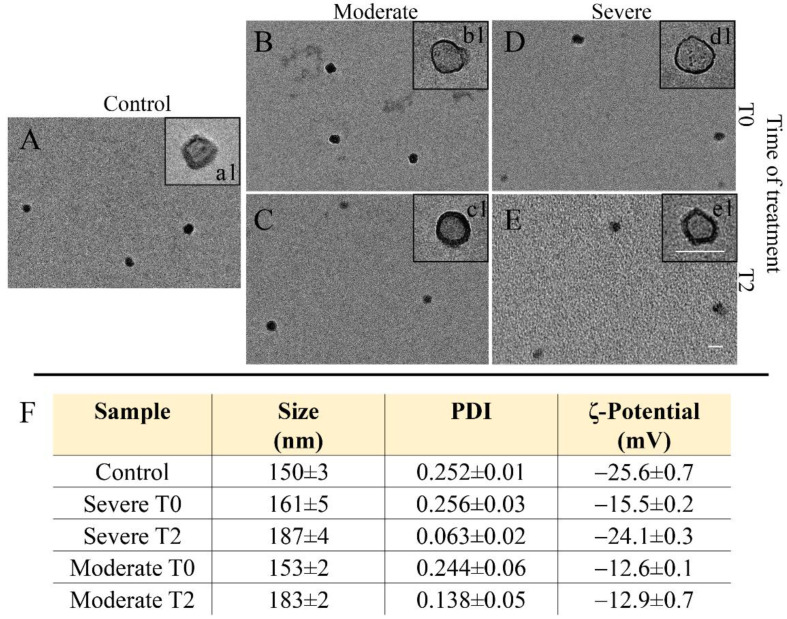
Exosomes freshly extracted from plasma of healthy subjects (20) and NAFLD-affected patients (95, 56 males and 39 females) with moderate (38) or severe (57) hepatic steatosis at recruitment time (T0) and after treatment (T2): representative TEM micrographs obtained with staining, for two increasing staining times, namely, 30 (a1–e1) and 60 s (**A**–**E**) (scale bar 100 nm); intensity-average hydrodynamic diameter and corresponding polydispersity index (PDI) determined by DLS and ζ-potential value. Mean ± SD are reported, *n* = 3 (**F**).

Exosomes were isolated from plasma of all the enrolled subjects, including the healthy subjects and the NAFLD-affected patients with moderate or severe hepatic steatosis at recruitment time (T0) and after 90 days of intervention (T2). DLS and TEM analysis and ζ-potential measurements were performed to characterize the freshly extracted exosomes in terms of size, size distribution, morphology, and surface charge (Figure 1). In their TEM representative micrographs, the presence of circular structures (Figure 1a1–e1) having a cup-shaped (Figure 1a1–e1) morphology, which is typically ascribed to surface dried exosomes, was observed. DLS and ζ-potential investigation resulted in a negative surface charge and an average hydrodynamic diameter of about 150 nm for all the isolated exosome samples (Figure 1F).

**Figure 2:**
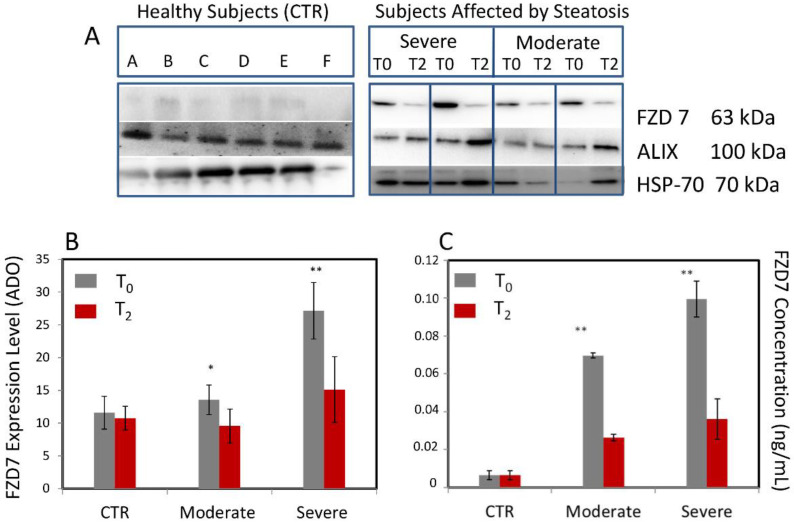
Exosomes extracted from plasma of all healthy subjects (20) and NAFLD-affected patients (95) with moderate (38) or severe (57) hepatic steatosis at recruitment time (T0) and after treatment (T2): (**A**) representative Western Blotting of FZD7, ALIX, and HSP-70 (molecular mass markers are indicated on the right); (**B**) semi-quantitative estimation, by Western blotting analysis and densitometry of protein bands, of relative FZD7 expression level; (**C**) quantitative analysis of FZD7 by ELISA assay. For semiquantitative analysis, the same total protein content (20 µg) was loaded and FZD7 bands were evaluated upon normalization with the corresponding housekeeping ALIX protein band for each sample. * *p* < 0.05 and ** *p* < 0.001 versus control.

The expression levels of the FZD7 expression were investigated by Western blotting analysis on the samples, containing the same total protein content, extracted from exosomes isolated from the plasma of all enrolled patients affected by NAFLD, with moderate and severe steatosis, before (T0) and after (T2) intervention. Controls were represented by the FZD7 expression levels in the protein content extracted from exosomes of the 20 healthy subjects at T0 time and after 90 days. Densitometry analysis was used to perform the semi-quantitative evaluation of the FZD7 expression level in the samples; normalization of the FZD7 band was carried out by using the HSP-70 protein band for each subject (Figure 2A,B). In Figure 2A, the presence of the two relevant marker proteins for exosomes, namely, ALIX and HSP-70, can be detected.

Semiquantitative analysis of the FZD7 expression levels performed on the control group provided an average value of 11.58 ± 2.51 ADO at T0 and 10.76 ± 1.82 ADO after 90 days (T2), thus indicating values of the FZD7 expression levels that can be considered normal in the healthy donors (Figure 2A,B). Semiquantitative analysis of the FZD expression levels performed on the NAFLD-affected subjects resulted in an average value of 13.55 ± 2.5 ADO at T0 and 9.56 ± 2.60 ADO at T2 for patients with moderate steatosis, while of 26.14 ± 4.30 ADO at T0 and 15.11 ± 5.0 ADO at T2 for patients with severe steatosis. A statistically significant increase of the FZD7 expression levels (versus control) was found in the protein content extracted by plasma-derived exosome of patients affected by NAFLD with moderate (** *p* < 0.005) and severe (* *p* < 0.0001) steatosis before intervention (T0), while a decrease was observed for the same patients after the administration of therapy (T2) (Figure 2A,B).

For comparison, ELISA assay was performed on protein content extracted from plasma-derived exosomes of the same healthy and NAFLD-affected subjects to quantitatively evaluate the FZD7 concentration (expressed in ng/mL) and assess the validity of the analytical method (Figure 2C). A significantly higher FZD 7 concentration (* *p* < 0.0001) in both categories of patients was observed at T0, while no significant difference was achieved after treatment (T2), with respect to controls.

Therefore, results of Western blotting analysis were confirmed by the outcome of the ELISA test, which is useful for a direct quantification of circulating FZD7 vehiculated by the exosomes. The ELISA approach results are useful, manageable, and applicable, even in clinical evaluations.

### 3.2. Characteristics of Participants

The characteristics of participants are reported in Table 1. No gender-related difference was observed. More than 50% of participants were 50 years or older, and 56.5% were males. Most subjects were ≥40 years old and male, with a varying FZD7 expression. Overall, FZD7 expression was higher for the NAFLD subjects in comparison with controls, while moderate NAFLD patients presented a lower FDZ7 expression than the severe ones. Older subjects tended towards higher levels of FZD7 expression than younger ones, and the higher the NAFLD severity, the higher the detected FZD7 expression. It was not possible to identify a particular pattern of FZD7 expression between sexes, and the FZD7 expression was randomly distributed among all intervention arms.

Other information regarding anthropometric information, lifestyle habits, co-morbidity, demographics, weight loss, etc., collected by the nutritionists, are reported in Tables S2–S4 in the Supplementary Information.

### 3.3. Effects of Diet, Lifestyle, Sex, and Age on FZD7 Expression

Results obtained by using ANOVA (repeated measures analysis of variance) are reported in Table 2. No main significant effect of NAFLD grade of severity and time of intervention on the FDZ7 expression was found. However, there was a significant modification effect between NAFLD severity and time in both moderate (β −3.99, 95% CI − 6.94; −1.05) and severe (β − 11.03, 95% CI − 13.52; −8.53) NAFLD.

**Figure 3:**
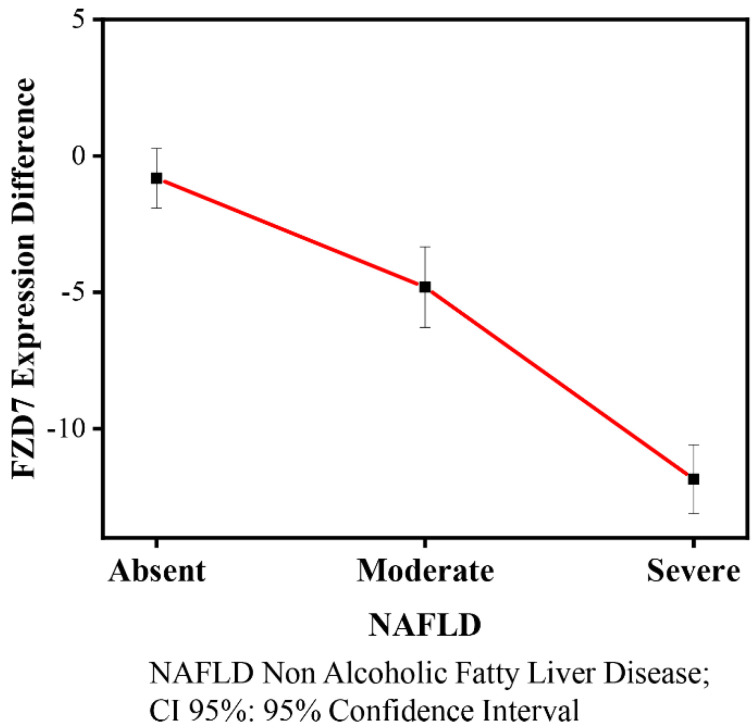
FZD7 expression and NAFLD contrasts (95% CI) by grade of severity. Number of healthy subjects (NAFLD absent) = 20, number of patients affected by moderate steatosis (NAFLD moderate) = 38, and severe steatosis (NAFLD Severe) = 57.

The contrasts of the FZD7 mean level expression among NAFLD severity (moderate and severe) and time (baseline and 90 days) are shown in Table 3 and graphically displayed in Figure 3. There were statistically significantly decreased FZD7 mean expression levels in moderate (−4.81, 95% CI −6.80; −2.83) and severe (−11.85, 95% CI 13.07; −10.62) NAFLD patients at the 90th day when compared with the reference category (NAFLD absent, baseline).

**Figure 4:**
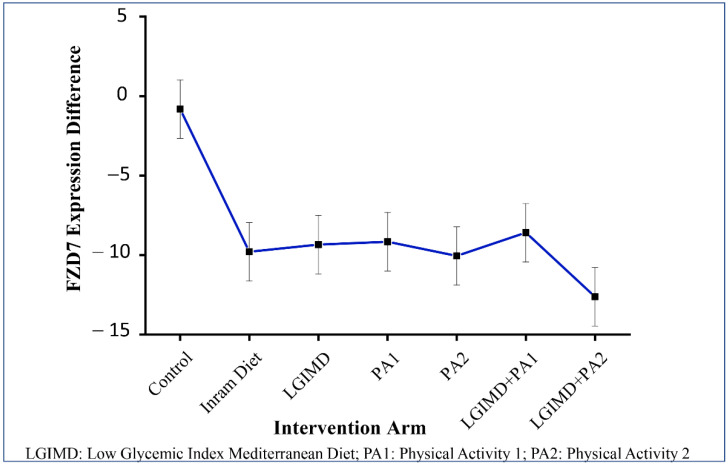
FDZ 7 expression and intervention arm: contrasts and 95% CI. The control group consisted of 20 subjects, the Inram diet group consisted of 16 subjects, the LGIMD group consisted of 17 subjects, the PA1 group consisted of 16 subjects, the PA2 group consisted of 14 subjects, the LGIMD + PA1 group consisted of 16 subjects, and the LGIMP + PA2 group consisted of 16 subjects.

To further probe the effect of different treatments on FZD7 expression levels, ANOVA including main effects for intervention arm and time and their modification effect was performed. Moreover, contrast among FZD7 mean expression differences by time and each intervention arm were explored. The results of these analyses are shown in Table 4 and Table 5. No significant effects of intervention and time is highlighted. However, when the modification effect between intervention arm and time on FDZ7 was considered, all combinations evidenced a statistically significant decrease ranging from −7.77 (95% CI −11.54; −3.99) for PA1 plus LGIMD to −11.79 (95% CI −15.64; −7.95) for PA2 plus LGIMD. Contrast analysis evidenced statistically significant negative differences of FDZ7 expression levels in all combinations of intervention arms and time. These results are graphically displayed in Figure 4.

### 3.4. FZD7 Expression in Liver Tissue Derived from NAFLD Patients

**Figure 5:**
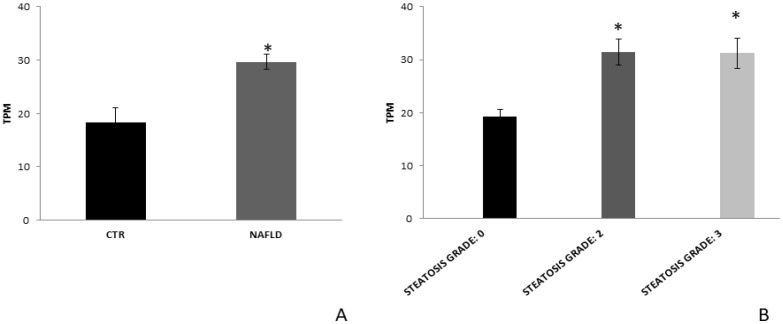
FZD7 mRNA expression in liver tissue from NAFLD patients. (**A**) FZD7 expression data were obtained from the analysis of RNA sequencing of liver tissue from the 72 patients with severe NAFLD and from the 6 controls, downloaded from Gene Expression Omnibus database (GSE130970). (**B**) FZD7 expression data in the same cohort of NAFLD patients divided according to steatosis grade, including the 42 patients with moderate NAFLD and 71 with severe NAFLD. Mean expression data were expressed in TPM (transcripts per million). * *p* < 0.05.

In order to further assess the FZD7 modulation in NAFLD patients, the gene expression profile was analyzed in liver tissue derived from 72 patients with NAFLD and from six histologically normal controls, downloaded from Gene Expression Omnibus database (GSE130970) [33]. FZD7 mRNA expression was found to be significantly upregulated compared to healthy controls (*p* < 0.05, Figure 5A). Furthermore, the grouping of patients according their steatosis grade revealed that FZD7 expression remained significantly upregulated in all the patient groups (*p* < 0.05, Figure 5B).

## 4. Discussion

Data obtained by Western blotting analysis of plasma-derived exosomes from NAFLD and healthy subjects were carefully analyzed by using a comprehensive statistical study. Despite the multifactorial nature of NAFLD, sedentary lifestyles, high carbohydrate intake, and insulin resistance are all external factors that contribute to NASH, an increasing aggressive form of NAFLD [34]. Very frequently, NAFLD evolves into NASH completely asymptomatic, and an effective monitoring even with the instruments now available turns out to be difficult [35]. In this regard, the identification and validation of novel and effective diagnostic and prognostic biomarkers are crucial for the management of this disease. Stearoyl-CoA desaturase (SCD) is an enzyme involved in the NAFLD progression; in normal conditions, it generates monounsaturated fatty acids (MUFAs) and usually contributes to cell growth, survival, differentiation, metabolic regulation, and signal transduction. Conversely, when overexpressed, SCD is involved in metabolic diseases such as diabetes and NAFLD, by regulating the canonical Wnt pathway through the activation of β-catenin and the consequent activation of hepatic stellate cells (HSCs), which support liver cell degeneration [36,37]. In particular, Wnt5a is involved in the liver degeneration, being overexpressed in liver fibrosis, which presents FZD2 and FZD7 as receptor [38,39]. The FZDs proteins have been found to be vehiculated by exosomes. In the case of liver injury, the FZD7 is upregulated or downregulated in the hepatic cells during hepatocellular degeneration [40]. Specifically, the inhibition of FZD7 with specific monoclonal antibody suppresses the cell proliferation and evolution of HCC [41,42]. Moreover, FZD7 has been implicated in other types of cancers, such as breast and lung cancers, causing fibrosis [43,44].

In this research, statistical analysis proved that the average expression of the FZD7, achieved by Western blotting analysis, in exosomes derived from plasma of NAFLD patients with moderate or severe steatosis was found to be significantly higher at baseline than healthy subjects, while the values were normalized after 90 days of specific interventions according to dietary modifications and/or associated with physical activity. It is well known that proper changes in lifestyle, such as dietary modifications (at reduced carbohydrates and fats intake), physical activity, and weight loss, can provide relevant benefits in NAFLD-affected subjects and even resolution of the pathology, depending on its severity [45,46]. NAFLD has been defined as a hepatic manifestation of metabolic syndrome, and several studies have identified genetic and epigenetic factors for their prognostic significance in follow-up studies [10]. Aerobic exercise is considered to be a useful intervention that can be used to prevent several chronic diseases [47]. Osella et al. observed that the modification of lifestyle, with the introduction of different correct diets and a specific physical activity program, resulted in a reduction of NAFLD scores, observing in particular the most efficient reduction was noticed for the intervention consisting of LGIMD associated with PA [21,48]. Furthermore, the benefit of a LGIMD diet, enriched in omega-6 PUFAs associated with PA, can be used in the prevention protocols for NAFLD, a chronic disease. Statistical analysis also demonstrated that, for the patients with severe steatosis, the FZD7 expression was higher than those found for subjects with moderate steatosis. Although all interventions were able to induce a significant decrease of FZD expression in plasma-derived exosomes from the NAFLD-affected participants, some interventions, namely, LGIMD + PA2, were found to be more efficacious than others to normalize the value of the FZD expression. Furthermore, the bioinformatic data obtained from the gene dataset concerning the evaluation of the FZD7 gene in tissues of patients with moderate (grade 2) and severe (grade 3) NAFLD downloaded from Gene Expression Omnibus database corroborated that the FZD7 mRNA expression in patients was found to be significantly upregulated according their steatosis grade when compared to healthy controls, thus confirming the usefulness of the protein FZD7 contained in the exosomes in the follow-up of the disease.

This study has several strengths and limitations. Strengths include the study design, the measured compliance to both dietary and PA interventions, and their controlled application, as well as an adequate sample size. Moreover, a well-validated assessment of the outcome such as FibroScan^®^ has been implemented. The applied intention-to-treat analytical strategy prevents the design from introducing bias related to non-adherence to the protocol to the prognosis; therefore, this RCT provides an unbiased assessment of treatment efficacy. It is worth noting that in this area, the most prevalent dietary pattern is the local version of the Mediterranean diet; then, a dilution bias could be present. Another limitation may be the duration of the intervention, which prevents wide application in the clinical field. Despite these described limitations, our study has allowed for the highlighting of the fact that downregulation of the FZD7 occurs by following all the tested interventions, and that LGIMD + PA2 resulted in being the most effective treatment.

In conclusion, the obtained results have indicated that exosomal FZD7 correlates with worsening of NAFLD and consistent improvements following different lifestyle interventions, suggesting a role for FZD7 as a potential non-invasive biomarker for NAFLD management.

## Figures and Tables

**Table 1:** Mean FZD7 expression by socio-demographic, intervention arm, and NAFLD severity. The control group consisted of 20 people, the Inram diet group consisted of 16 subjects, the LGIMD group consisted of 17 subjects, the PA1 group consisted of 16 subjects, the PA2 group consisted of 14 subjects, the LGIMD + PA1 group consisted of 16 subjects, and the LGIMP + PA2 group consisted of 16 subjects.

	NAFLD							
	Absent		Moderate		Severe		Total	
	Mean	SE	Mean	SE	Mean	SE	Mean	SE
Age (categorical, years)								
<40 (*n* = 19)	12.30	(1.05)	9.85	(0.97)	11.56	(1.18)	11.71	(0.73)
40–49 (*n* = 36)	10.51	(0.36)	9.21	(0.65)	16.35	(1.32)	13.44	(0.97)
≥50 (*n* = 60)	9.34	(0.97)	9.87	(1.35)	15.02	(0.97)	13.20	(0.79)
Total (*n* = 115)	10.76	(0.65)	9.56	(0.70)	15.11	(0.73)	13.05	(0.53)
Sex								
Male (*n* = 65)	10.33	(0.85)	10.21	(1.06)	14.12	(1.01)	12.43	(0.70)
Female (*n* = 50)	11.19	(0.97)	8.49	(0.31)	16.14	(1.03)	13.77	(0.80)
Total (*n* = 115)	10.76	(0.65)	9.56	(0.70)	15.11	(0.73)	13.05	(0.53)
Intervention								
Control (*n* = 20)	10.76	(0.65)					10.76	(0.65)
Inram diet (*n* = 16)			8.05	(0.46)	16.47	(2.02)	14.22	(1.77)
LGIMD (*n* = 17)			10.45	(0.58)	15.56	(1.74)	14.19	(1.41)
PA1 (*n* = 16)			12.90	(4.04)	14.91	(1.48)	14.51	(1.45)
PA2 (*n* = 14)			9.27	(1.38)	15.61	(2.44)	14.45	(2.14)
LGIMD + PA1 (*n* = 16)			8.66	(1.10)	14.67	(1.86)	12.04	(1.37)
LGIMD + PA2 (*n* = 16)			9.43	(0.49)	13.48	(1.08)	12.40	(0.93)
Total (*n* = 115)	10.76	(0.65)	9.56	(0.70)	15.11	(0.73)	13.05	(0.53)

**Table 2:** Repeated measures analysis of variance. Expected mean FZD7 expression and NAFLD scores (as assessed by CAP value) by time and NAFLD severity. NutriAtt Trial, Castellana Grotte, 2015–2016. Number of healthy subjects (absent) = 20, number of patients affected by moderate steatosis = 38, and severe steatosis = 57.

FZD7	β	SE	*p*-Value	(95% CI)
NAFLD				
Absent *n* = 20	0			
Moderate *n* = 38	−9.40	9.87	0.34	(−28.98; 10.18)
Severe *n* = 57	3.27	6.58	0.62	(−9.78; 16.33)
Time				
Baseline	0			
90 days	−0.82	1.09	0.46	(−2.99; 1.36)
NAFLD × Time				
Moderate × 90 days	−3.99	1.48	0.008	(−6.94; −1.05)
Severe × 90 days	−11.03	1.26	0.000	(−13.52; −8.53)

**Table 3:** Repeated measures analysis of variance. Contrast of FZD7 expression between NAFLD severity and time exosomes extracted from plasma of healthy subjects and NAFLD-affected patients. NutriAtt Trial, Castellana Grotte, 2015–2016. Number of healthy subjects (NAFLD absent) = 20, number of patients affected by moderate steatosis (NAFLD moderate) = 38, and severe steatosis (NAFLD Severe) = 57. * *p* = 0.005.

FZD7	Contrast (CI 95%)
(90daysVs baseline) NAFLD absent	−0.82 (−2.99; 1.35)
(90days Vs baseline) NAFLD Moderate	−4.81 (−6.80; −2.83) *
(90days Vs baseline) NAFLD Severe	−11.85 (−13.07; −10.62)

**Table 4:** Repeated measures analysis of variance. Effect of treatments on FZD7a expressions by intervention arms and time. NutriAtt Trial, Castellana Grotte, 2015–2016 ^a^ FDZ7; ^a^ LGIMD: low glycemic index Mediterranean diet; ^b^ PA1: aerobic activity program; ^c^ PA2: combined activity program; ^d^ PA1 + LGIMD; ^e^ PA2 + LGIMD. The control group consisted of 20 subjects, the Inram diet group consisted of 16 subjects, the LGIMD group consisted of 17 subjects, the PA1 group consisted of 16 subjects, the PA2 group consisted of 14 subjects, the LGIMD + PA1 group consisted of 16 subjects, and the LGIMP + PA2 group consisted of 16 subjects. * *p* = 0.000.

FDZ7	β	SE	*p*-Value	(CI 95%)
Working arms				
Control subjects (*n* = 20)	0			
Inram diet (*n* = 16)	12.25	12.56	0.33	(−12.67; 37.17)
LGIMD ^a^ (*n* = 17)	19.22	11.77	0.10	(−4.14; 42.58)
PA1 ^b^ (*n* = 16)	12.04	9.02	0.18	(−5.86; 29.93)
PA2 ^c^ (*n* = 14)	31.53	15.60	0.04	(−0.58; 62.48)
PA1 + LGIMD ^d^ (*n* = 16)	12.70	16.56	0.44	(−20.16; 45.56)
PA2 + LGIMD ^e^ (*n* = 16)	8.39	11.77	0.47	(−14.96; 31.76)
Time				
Baseline	0			
90 days	−0.82	1.27	0.52	(−3.34; 1.69)
(90 days vs. baseline) Inram diet	−8.97	1.93	0.000	(−12.82; −5.13) *
(90 days vs. baseline) LGIMD ^a^	−8.53	1.93	0.000	(−12.37; −4.68) *
(90 days vs. baseline) PA1 ^b^	−8.34	1.93	0.000	(−12.18; −4.49) *
(90 days vs. baseline) PA2 ^c^	−9.23	2.13	0.000	(−13.45; −5.01) *
(90 days vs. baseline) PA1 + LGIMD ^d^	−7.77	1.90	0.000	(−11.54; −3.99) *
(90 days vs. baseline) PA2 + LGIMD ^e^	−11.79	1.94	0.000	(−15.64; −7.95) *

**Table 5:** Measures analysis of variance. Effect of treatments on FDZ7a expression. Contrasts by time. NutriAtt Trial, Castellana Grotte, 2015–2016 ^a^ FDZ7; ^a^ LGIMD: low glycemic index Mediterranean diet; ^b^ PA1: aerobic activity program; ^c^PA2: combined activity program; ^d^ PA1 + LGIMD; ^e^ PA2 + LGIMD. * *p*-value < 0.05. The control group consisted of 20 people, the Inram diet group consisted of 16 subjects, the LGIMD group consisted of 17 subjects, the PA1 group consisted of 16 subjects, the PA2 group consisted of 14 subjects, the LGIMD + PA1 group consisted of 16 subjects, and the LGIMP + PA2 group consisted of 16 subjects.

FDZ7	Contrast	(CI 95%)
(90 days vs. baseline) control subjects *n* = 20	−0.82	(−3.34; 1.69)
(90 days vs. baseline) Inram diet *n* = 16	−9.79	(−12.70; −6.889) *
(90 days vs. baseline) LGIMD ^a^ *n* = 17	−9.34	(−12.25; −6.44) *
(90 days vs. baseline) PA1 ^b^ *n* = 16	−9.16	(−12.07; −6.26) *
(90 days vs. baseline) PA2 ^c^ *n* = 14	−10.05	(−13.44; −6.66) *
(90 days vs. baseline) PA1 + LGIMD ^d^ *n* = 16	−8.59	(−11.40; −5.78) *
(90 days vs. baseline) PA2 + LGIMD ^e^ *n* = 16	−12.62	(−15.52; −9.71) *

## Data Availability

The data used to support the findings of this study are included within the article.

## Supplementary Materials

The following are available online at https://www.mdpi.com/article/10.3390/nu14061133/s1. Table S1: Post-estimation analysis by using Bonferroni Test, show statistical significative decrease value for CAP. Table S2: Adherence to phisical activity programs by working arm, gender and week from enrollment. NutriAtt Trial, Castellana Grotte 2015–2016. Table S3: Mediterranean adequacy Index by Gender, Age and week. NutriAtt trial. Castellana Grotte, 2015–2016. Table S4: Main characteristics of partecipants by interventation arm and time. NutriAtt trial, Castellana Grotte 2015–2016.
