# Supplementary material for: Exosomal FZD-7 Expression Is Modulated by Different Lifestyle Interventions in Patients with NAFLD
Source: Nutrients. 2022 Mar 8;14(6):1133. doi: 10.3390/nu14061133 (PMC8950750; doi:10.3390/nu14061133)
Supplement: Supplementary file 1 [file nutrients-14-01133-s001.zip › nutrients-1580491-supplementary.pdf]

nutrients

Supplementary Information

Exosomal FZD-7 Expression Is Modulated by Different Lifestyle Interventions in Patients with NAFLD

Maria Principia Scavo[1*], Nicoletta Depalo[2*], Federica Rizzi[2,3], Livianna Carrieri[1], Grazia Serino[4], Isabella Franco[5], Caterina Bonfiglio[5], Pasqua Letizia Pesole[6], Raffaele Cozzolongo[7], Vito Gianuzzi[7], Maria Lucia Curri[2,3], Alberto Ruben Osella[5§] and Gianluigi Giannelli[8§]

Table S1. Post-estimation analysis by using Bonferroni Test, show statistical significative decrease value for CAP

DIET	β	SE	95%CI
(Control#1)vs(Control#0)	-0.82	(1.27)	(-5.35;3.71)
(LGIMD#1) vs (LGIMD#0)	-9.35	(1.46)	(-14.57; -4.12)
(PA1#1) vs (PA1#0)	-9.16	(1.46)	(-14.39; -3.93)
(PA2#1) vs (PA2#0)	-10.05	(1.71)	(-16.16; -3.95)
(LGIMD+PA1#1)vs(LGIMD+PA1#0)	-8.59	(1.42)	(-13.65; -3.53)
(LGIMD+PA2#1)vs(LGIMD+PA2#0)	-12.62	(1.46)	(-17.85; -7.39)

Table S2: Adherence to phisical activity programs by working arm, gender and week from enrollment. NutriAtt Trial, Castellana Grotte 2015-2016

		PA1[a]		PA2[b]		LGIMD+PA1[c]		LGIMD+PA2[d]	
		Male	Female	Male	Female	Male	Female	Male	Female
Weeks		Mean (SD)	Mean (SD)	Mean (SD)	Mean (SD)	Mean (SD)	Mean (SD)	Mean (SD)	Mean (SD)
	Time	96.5 (9.70)	100 (0)	100 (0)	100 (0)	97.8 (8.60)	93.7 (12.40)	100 (0)	100 (0)
3	Intensity	101.6 (3.81)	100.5 (1.54)	100.1 (0.61)	100 (0)	99.9 (1.22)	100 (0)	99.9 (0.45)	99.4 (2.41)
	Load	N/A*	N/A*	100 (0)	100 (0)	N/A*	N/A*	10 (0)	100 (0)
	Time	92.4 (15.8)	89.6 (19.8)	100 (0)	100 (0)	100 (0)	100 (0)	100 (0)	97.4 (9.24)
4	Intensity	101.5 (3.20)	100.5 (1.54)	100.1 (0.61)	100 (0)	100.3 (0.63)	100 (0)	99.9 (0.51)	99.4 (2.41)
	Load	N/A*	N/A*	100 (0)	100 (0)	N/A*	N/A*	100 (0)	100 (0)
	Time	94.4 (12.9)	95.8 (11.8)	95.8 (11.4)	94.4 (13.6)	100 (0)	100 (0)	100 (0)	94.9 (12.5)
5	Intensity	101.0 (2.32)	100.1 (0.28)	100.1 (0.61)	100 (0)	100.6 (1.56)	100 (0)	100.1 (0.40)	98.9 (2.81)
	Load	N/A*	N/A*	100 (0)	100 (0)	N/A*	N/A*	100 (0)	100 (0)
	Time	94.2 (11.5)	100 (0)	93.7 (13.4)	94.4 (13.6)	100 (0)	100 (0)	100 (0)	97.4 (9.24)
6	Intensity	100.5 (3.07)	100.1 (0.28)	99.9 (0.41)	100 (0)	100.9 (1.84)	100 (0)	100.1 (0.40)	98.9 (2.81)
	Load	N/A*	N/A*	100 (0)	100 (0)	N/A*	N/A*	100 (0)	100 (0)
	Time	93.0 (13.2)	92.0 (22.6)	95.8 (11.4)	94.4 (13.6)	97.8 (8.60)	87.5 (24.8)	100.0 (0)	92.3 (20.0)
7	Intensity	100.6 (1.53)	100.1(0.28)	100 (0)	100 (0)	100.5 (1.03)	100.0 (0)	100.1 (0.40)	98.9 (2.81)
	Load	N/A*	N/A*	100 (0)	100 (0)	N/A*	N/A*	100 (0)	100 (0)
	Time	88.2 (19.3)	91.7 (15.4)	95.8 (16.7)	100 (0)	100 (0)	83.3 (27.9)	100 (0)	97.4 (9.24)
8	Intensity	100.5 (1.66)	100.1 (0.28)	100 (0)	100 (0)	100.6 (1.08)	100 (0)	100.1 (0.40)	98.9 (2.81)
	Load	N/A*	N/A*	100 (0)	100 (0)	N/A*	N/A*	100 (0)	100 (0)
	Time	93.4 (16.1)	94.8 (11.7)	93.3 (13.8)	91.7 (16.7)	97.4 (9.24)	88.9 (17.2)	97.9 (8.33)	97.4 (9.24)
9	Intensity	99.6 (1.26	100 (0)	100.2 (0.73)	100 (0)	100.1 (1.07)	100 (0)	100.2 (0.79)	98.6 (3.43)
	Load	N/A*	N/A*	100 (0)	100 (0)	N/A*	N/A*	100 (0)	100 (0)
10	Time	98.4 (5.09)	84.5 (19.5)	93.3 (13.8)	91.7 (16.7)	97.4 (9.24)	94.4 (13.6)	97.9 (8.33)	94.8 (12.5)

[a]PA1: Physical Activity 1 based on the Aerobic Activity Program; [b]PA2: Physical Activity 2 based on the combination of Aerobic Activity Program and Resistance Training; [c]PA1+LGIMD: Low Glycemic Index Mediterranean Diet; [d]PA2+LGIMD: Low Glycemic Index Mediterranean Diet;*N/A= Not Applicable

Table S3: Mediterranean adequacy Index by Gender, Age and week. NutriAtt trial. Castellana Grotte, 2015-2016

		Male			Female		
		3rd week	6th week	9th week	3rd week	6th week	9th week
**Age (yrs)**	**Diet**	Median (IQR)	Median (IQR)	Median (IQR)	Median (IQR)	Median (IQR)	Median (IQR)
**<40**	**CD[a]**	2.3 (0.9:12.5)	2.3 (0.9:12.5)	3.5 (0.9;12.5)	7.8 (0.9;8.5)	7.8 (0.9;8.5)	0.9 (0.9;8.5)
	**LGMID[b]**	4.6 (1.8;25.1)	4.6 (1.8;25.1)	6.9 (1.8;25.1)	15.7 (1.8;17.0)	15.7 (1.8;17.0)	1.9 (1.8;17.0)
**40-49**	**CD[a]**	4.7 (2.0;9.4)	4.7 (2.6;15.2)	4.9 (2.6;15.2)	2.1 (0.9;3.3)	2.1 (0.9;3.3)	2.1 (1.6;3.3)
	**LGMID[b]**	9.5 (4.0;18.8)	9.5 (5.7;30.5)	9.7 (5.7;30.5)	3.6 (1.9;6.5)	3.6 (1.9;6.5)	3.6 (3.2;6.5)
**≥50**	**CD[a]**	4.7 (3.4;12.3)	5.2 (3.4;12.4)	4.7 (3.7;12.4)	5.9 (3.6;9.6)	5.9 (3.6;9.6)	5.9 (3.6;9.0)
	**LGMID[b]**	9.4 (5.8;24.6)	10.5 (5.4;26.4)	9.4 (5.8;26.4)	11.8 (7.4;19.1)	11.8 (7.4;19.1)	11.8 (7.4; 7.9)

[a]CD: Control Diet based on CREA-AN guidelines; [b]LGIMD: Low Glycemic Index Mediterranean Diet

Table S4: Main characteristics of partecipants by interventation arm and time. NutriAtt trial, Castellana Grotte 2015-2016

	Control	[a]CD	[b]LGIMD	[c]PA1	[d]PA2	[e]LGIMD+PA1	[f]LGIMD+PA2	*p-value*
N	20	16	17	16	14	16	16	
Age at Enrollment (yrs)*	45,35 (16,38)	53,63 (8,81)	56,82 (9,58)	53,44 (9,56)	45,79 (9,82)	49,31 (9,49)	50,25 (11,00)	0.029
Age (categorical, yrs)**								
<40	8 (40%)	0 (0%)	0 (0%)	2 (13%)	4 (29%)	2 (13%)	3 (19%)	0.003
40-49	4 (20%)	7 (44%)	3 (18%)	2 (13%)	6 (43%)	9 (56%)	5 (31%)	
≥50	8 (40%)	9 (56%)	14 (82%)	12 (75%)	4 (29%)	5 (31%)	8 (50%)	
Sex**								
Male	10 (50%)	7 (44%)	10 (59%)	9 (56%)	9 (64%)	9 (56%)	11 (69%)	0.84
Female	10 (50%)	9 (56%)	7 (41%)	7 (44%)	5 (36%)	7 (44%)	5 (31%)	
Smoke**								
Never/Former	16 (80%)	12 (92%)	12 (80%)	11 (79%)	7 (64%)	11 (85%)	10 (83%)	0.76
Current	4 (20%)	1 (8%)	3 (20%)	3 (21%)	4 (36%)	2 (15%)	2 (17%)	
Education**								
Primary School	4 (20%)	0 (0%)	2 (13%)	3 (21%)	5 (45%)	5 (38%)	3 (23%)	0.15
Secondary School	3 (15%)	0 (0%)	1 (7%)	0 (0%)	0 (0%)	1 (8%)	0 (0%)	
High School	4 (20%)	7 (54%)	6 (40%)	4 (29%)	1 (9%)	1 (8%)	2 (15%)	
Graduate	9 (45%)	6 (46%)	6 (40%)	7 (50%)	5 (45%)	6 (46%)	8 (62%)	
Marital Status**								
Single	7 (35%)	1 (8%)	0 (0%)	1 (7%)	2 (20%)	2 (15%)	1 (8%)	0.11
Married/Coupled	12 (60%)	12 (92%)	14 (100%)	13 (93%)	8 (80%)	9 (69%)	10 (83%)	
Separated/Divorced	1 (5%)	0 (0%)	0 (0%)	0 (0%)	0 (0%)	2 (15%)	1 (8%)	
Diabetes**								
No	17 (89%)	14 (100%)	12 (80%)	15 (94%)	13 (100%)	9 (69%)	11 (92%)	0.11
Yes	2 (11%)	0 (0%)	3 (20%)	1 (6%)	0 (0%)	4 (31%)	1 (8%)	
Dyslipidemia **								
No	17 (89%)	10 (71%)	8 (53%)	12 (75%)	10 (77%)	12 (86%)	9 (75%)	0.32
Yes	2 (11%)	4 (29%)	7 (47%)	4 (25%)	3 (23%)	2 (14%)	3 (25%)	
Hypertension**								
No	16 (84%)	10 (71%)	6 (40%)	10 (63%)	10 (77%)	10 (71%)	7 (58%)	0.18
Yes	3 (16%)	4 (29%)	9 (60%)	6 (38%)	3 (23%)	4 (29%)	5 (42%)	
[g]Ratiofzd7*								
Baseline	11,58 (2,51)	24,48 (7,71)	23,73 (5,87)	23,53 (5,74)	24,70 (6,45)	20,63 (6,48)	25,04 (7,69)	<0.001
Three months	10,76 (2,97)	14,22 (7,10)	14,19 (5,66)	14,51 (5,80)	14,45 (7,44)	12,04 (5,66)	12,40 (3,70)	0.30
[h]DBP (mmHg) *								
Baseline	116,50 (17,25)	124,69 (12,58)	131,56 (21,27)	122,50 (10,00)	116,15 (10,44)	124,06 (14,52)	129,38 (17,31)	0.038
Three months	114,00 (14,65)	119,55 (6,50)	123,08 (13,16)	120,00 (7,56)	113,00 (9,49)	117,50 (10,87)	123,00 (14,37)	0.19
[i]SBP (mmHg) *								
Baseline	76,75 (7,66)	80,63 (9,46)	84,06 (11,14)	79,06 (5,23)	78,46 (4,27)	80,63 (6,02)	84,38 (7,27)	0.041
Three months	77,65 (5,80)	79,09 (7,01)	77,69 (10,92)	81,25 (6,41)	77,00 (6,75)	79,00 (6,15)	80,67 (6,78)	0.77
Weight (Kg) *								
Baseline	66,89 (15,44)	91,46 (14,00)	89,35 (13,55)	87,00 (14,16)	90,15 (8,60)	91,00 (14,31)	102,64 (20,05)	<0.001
Three months	66,55 (14,92)	88,67 (15,32)	85,88 (13,55)	84,76 (13,85)	88,55 (8,38)	85,94 (12,85)	95,69 (19,10)	<0.001
[l]BMI (kg/m^2) *								
Baseline	23,86 (4,56)	33,47 (4,52)	32,49 (4,00)	31,28 (3,24)	31,36 (3,49)	33,96 (4,53)	35,80 (5,66)	<0.001
Three months	23,86 (4,56)	31,88 (4,84)	30,28 (4,22)	30,02 (3,38)	30,75 (3,68)	32,45 (4,00)	33,38 (5,31)	<0.001
Triglycerides (mmol/L) *								
Baseline	0,73 (0,44)	1,04 (0,45)	1,62 (1,11)	1,65 (1,10)	1,27 (0,69)	1,74 (1,21)	1,53 (0,73)	0.005
Three months	0,69 (0,36)	0,93 (0,28)	1,42 (0,81)	1,58 (0,55)	1,23 (0,65)	1,18 (0,57)	1,01 (0,42)	<0.001
Total Cholesterol (mmol/L) *								
Baseline	4,36 (0,51)	5,17 (1,14)	5,19 (1,34)	4,93 (0,98)	5,18 (1,05)	5,35 (1,02)	5,01 (0,90)	0.085
Three months	4,16 (0,52)	5,16 (0,78)	4,73 (1,12)	5,31 (0,78)	5,39 (1,08)	4,94 (1,13)	4,68 (1,01)	0.008

[a]CD: Control Diet based on CREA-AN guidelines; [b]LGIMD: Low Glycemic Index Mediterranean Diet; [c]PA1: Physical Activity 1 based on the Aerobic Activity Program; [d]PA2: Physical Activity 2 based o[illegible] the combination of Aerobic Activity Program and Resistance Training;[e]PA1+LGIMD; [f]PA2+LGIMD; [g]Ratiofzd7 ; [h]DBP: Diastolic Blood Pressure; [i]SBP Systolic Blood Pressure; [l]BMI: Body Mass Index [m]HDL: High Density Lipoprotein Cholesterol ;[n]ALT: Alanine Aminotrasferase; [o]AST: Aspartate Aminotransferase; [p]HOMA-IR: Homeostasis model assessment for insulin resistance; [q]CAP: Controlled Attenuatio[illegible] Parameter FibroScan®;

Cells reporting subject characteristics contain * Mean±(SD). **Number. (Percentage) Percentages calculated per column***Median (IQR)

Brochure of prescribed diets

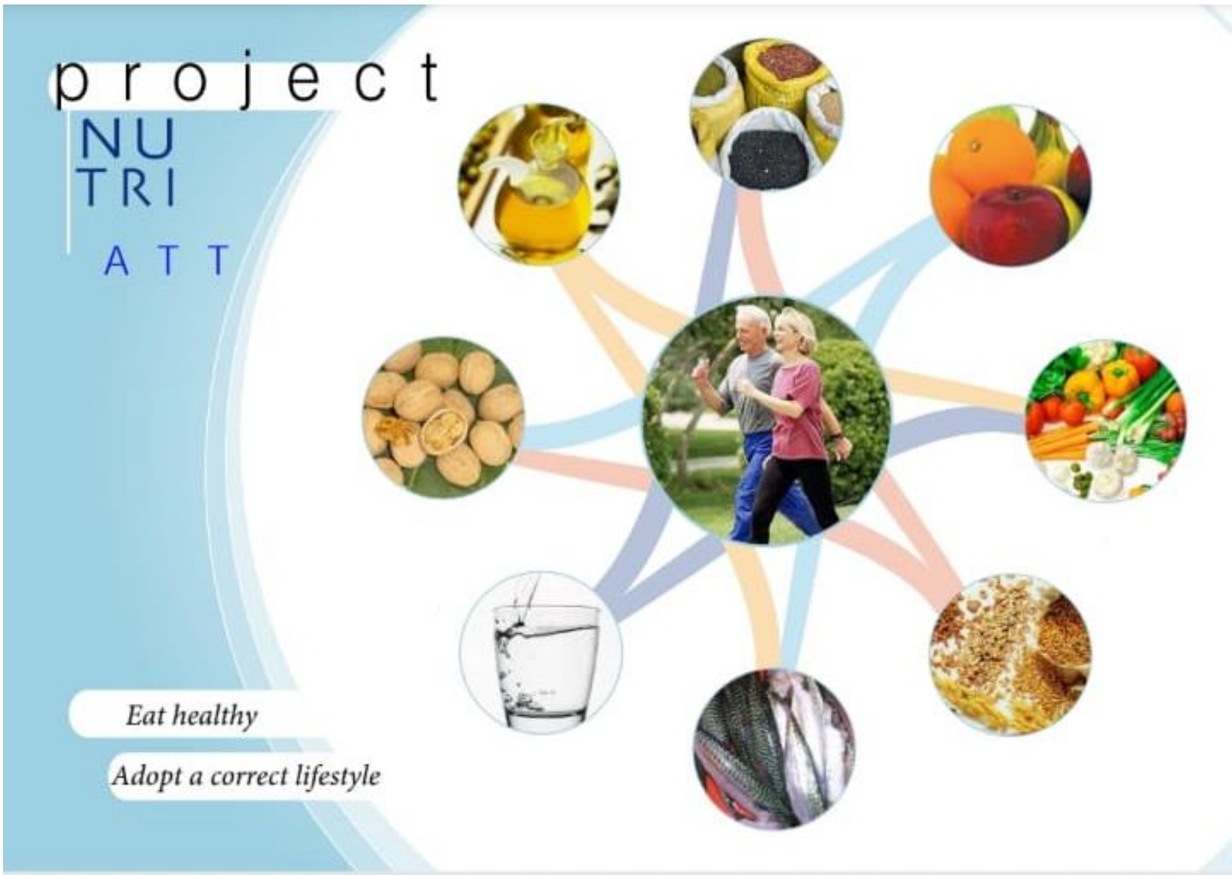

Introduction

Your health is in your hands; you build it each day when you go to the supermarket and choose one food rather than another one, when you cook a lunch or a dinner. In these moments you do dietary choices which influence your health. A wrong diet can determine the appearance of risk factors for cardiovascular diseases and cancer. One of the steps of these diseases is the Non-alcoholic Fatty Liver Disease (NAFLD) (fat liver).

We found that your clinical, laboratory and instrumental data , which came from the NutriEP study you participate in, evidenced that you have NAFLD and then you are at a greater risk to develop cardiovascular disease or tumors (the principal causes of death in Western World).

The Laboratory of Epidemiology and Biostatistics of the IRCCS Saverio de Bellis from Castellana Grotte (Bari) has started up the research project "NutriEpa: EFFECT OF A LOW GLYCEMIC INDEX MEDITERRANEAN DIET ON NON ALCOHOLIC FATTY LIVER DISEASE. A RANDOMIZED CONTROLLED CLINICAL TRIAL" to evaluate the efficacy of the best diet to treat NAFLD".

So, we invite you to participate in this project which will help us to understand the efficacy of the diet to treat NAFLD. Dietary advices described in the following pages are those of Low Glycemic Index Mediterranean Diet and we ask you to follow them. Furthermore we ask you to record your daily diet in this dietary record as long as the duration of the study. This will permit us to measure adherence level to the diet we gave you.

WATCH THE SEMAPHORE!

CHECK YOUR DIETARY CHOICES

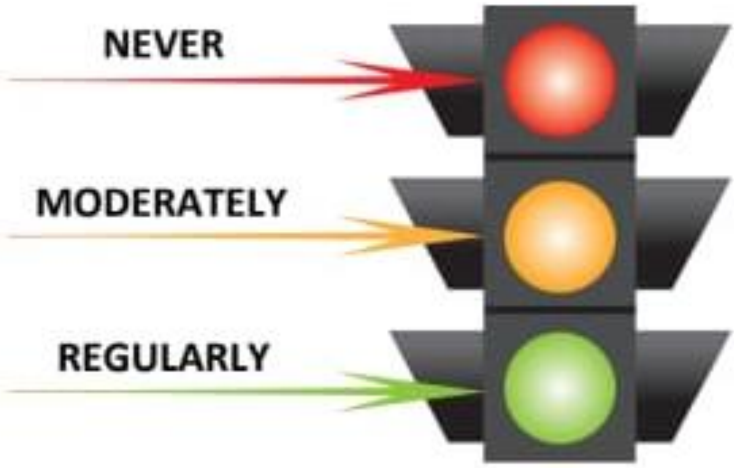

Foods choice and the frequency of their consumption is fundamental to set a correct dietary programm, which day after day should be followed by everyone to keep a satisfying state of health. To direct foods choice towards those which best represent the **LOW GLYCAEMIC INDEX MEDITERRANEAN DIET**, we marked with **GREEN** colour the foods to consume regularly, with **YELLOW** those to consume moderately and with **RED** those to avoid completely.

REGULARLY
1- Raw vegetables (lettuce, tomatoes, cucumbers, celery, carrots, radishes, etc.)
2- Steamed or boiled vegetables (beets, turnips, chicory, cauliflower, broccoli, etc.)
45- Dried pulses without something else (lentils, chickpeas, beans, broad beans, soybeans)
46- Own fresh vegetables without something else (peas, beans, green beans)
12- Whole wheat pasta with pulses
13- Whole wheat pasta with vegetables
18- Brown rice with pulses
19- Brown rice with vegetables
42- Fish (anchovies, sardines, mackerel, etc.)
40- Molluscs and crustaceans
43- Cod, swordfish, fresh tuna
66- Bass bream salmon
44- Canned tuna
28- Extra virgin olive oil (raw)
5- Unsweetened fresh fruit (apples, pears, oranges, grapefruit, kiwi, peaches, etc.)
8- Unsweetened nuts (walnuts, almonds)
65- Coffee (without sugar or with artificial sweetener)
52 -Natural water

MODERATELY	max
30- Milk and yogurt	1 time/daily
56- Whole wheat biscuits	1 time/daily (3 biscuits)
31- Cheese (parmigiano, cheese, etc.)	2 times/weekly
32- Dairy products (cheese, smoked cheese, cottage cheese)	2 times/weekly
33- Eggs	2 time/weekly (1 egg)
34- White meat (chicken, turkey, rabbit)	2 times/weekly
25- Potatoes (boiled only)	1 time/weekly
22- Whole wheat bread	2 times/daily (a slice)
15- Whole wheat pasta with simple sauce	2 times/weekly (max 80 gr)
4- Sugary fruit (bananas, persimmons, grapes)	1 time/daily (max 1 piece)
62- Dense pure honey	1 time/daily (a teaspoon)
49-wine	2 times/daily (a glass

NEVER
11- Not whole wheat pasta
17- Not whole wheat rice
21- White bread
53- Crackers, pretzels, bread sticks, bread and breakfast cereals
47- Pizza
35- Red meat (beef, veal, pork, horse, etc.)
36- Canned meat
37- Stew
38- Sausages (raw or cooked ham, dried beef, bacon, salami, mortadella, etc.)
41- Farmed fish
26- French fries or baked
67- Fry
29- Butter, margarine, mayonnaise, cream
9- Fruit in syrup and candied fruit
7- Sweet dried fruit (dried figs, dried dates, prunes, raisins)
63- Sugar
60- Candies and chocolates
61- Jams
57- Cakes, sweets, pastries
58- Croissants, pastries and snacks
54- Dry biscuits, toasts
55- Biscuits, shortbread
59- Ice-cream and water ice
51- Alcoholic beverages and spirits (brandy, grappa, liqueurs, whiskey)
50- Carbonated/Non carbonated soft drinks (orange juice, coca-cola, fruit juice, etc.)
48- Beer

Your Dietary Record

Instructions

- It's fundamental to complete the Dietary Record each day immediately after the meal or as soon as possible.
- The Dietry Record is composed of a series of pages, one for each week. In each weekly page, days from Monday to Sunday are divided in breakfast, lunch, dinner and snack.
- Record all the foods you eat and what you drink during the day (Sunday and holydays included).
- Registration consists in taking note, in correspondence to the meal, the identification number of the food/drink or of the food group consumed belonging to the list we enclosed. If the food you ate isn't included in the list, just write its name in the place reserved to the corresponding meal.

Example:

Here below it is shown an hypothetical day of Mr Rossi's Dietary Record.

After each meal Mr Rossi registered what he ate and drank, so he wrote in the box of the specific meal the correponding number of the food he consumed (by checking the foods list).

At breakfast he drank a cup of milk and coffee without sugar and he ate an apple.

	BREAKFAST	Snack	LUNCH	Snack	DINNER	Snack	PHYSICAL ACTIVITY
MONDAY	30+65<br>5						☐ None<br>☐ Walk<br>☐ Housework<br>☐ Training<br>☐ Other (specify what)

At lunch he had a plate of lentils (without pasta) seasoned with raw extra-virgin olive oil, a cod, a plate of raw unseasoned vegetables and he drank two glasses of wine and a glass of natural water.

	BREAKFAST	Snack	LUNCH	Snack	DINNER	Snack	PHYSICAL ACTIVITY
MONDAY			45 + 28<br>43, 1<br>49 + 49<br>52				☐ None<br>☐ Walk<br>☐ Housework<br>☐ Training<br>☐ Other (specify what)

In the afternoon he had an apple and drank a coffee without sugar.

	BREAKFAST	Snack	LUNCH	Snack	DINNER	Snack	PHYSICAL ACTIVITY
MONDAY				5<br>65			☐ None<br>☐ Walk<br>☐ Housework<br>☐ Training<br>☐ Other (specify what)

At dinner he had a plate of minestrone soup with pasta and seasoned with raw extra-virgin olive oil, a slice of whole bread, a pear and drank two glasses of natural water.

	BREAKFAST	Snack	LUNCH	Snack	DINNER	Snack	PHYSICAL ACTIVITY
MONDAY	30 + 65<br>5		45 + 28<br>43, 1<br>49 + 49<br>52	5<br>65	Minestrone soup<br>+ 28<br>22<br>52 + 52		☐ None<br>☒ Walk<br>☐ Housework<br>☐ Training<br>☐ Other (specify what)

In the last column, referred to the physical activity carried out during the day, he put a cross in correspondence to the entry "walk".

SURNAME ______ LAST NAME ______ CODE ______
MONTH ______ WEEK from ______ to ______

	BREAKFAST	Snack	LUNCH	Snack	DINNER	Snack	PHYSICAL ACTIVITY
Monday							☐ None<br>☐ Walk<br>☐ Housework<br>☐ Training<br>☐ Other (specify what) ______
Tuesday							☐ None<br>☐ Walk<br>☐ Housework<br>☐ Training<br>☐ Other (specify what) ______
Wednesday							☐ None<br>☐ Walk<br>☐ Housework<br>☐ Training<br>☐ Other (specify what) ______
Thursday							☐ None<br>☐ Walk<br>☐ Housework<br>☐ Training<br>☐ Other (specify what) ______
Friday							☐ None<br>☐ Walk<br>☐ Housework<br>☐ Training<br>☐ Other (specify what) ______
Saturday							☐ None<br>☐ Walk<br>☐ Housework<br>☐ Training<br>☐ Other (specify what) ______
Sunday							☐ None<br>☐ Walk<br>☐ Housework<br>☐ Training<br>☐ Other (specify what) ______

Ministry of
Health

DE BELLIS

Research Laboratories
I.R.C.C.S. "Saverio De Bellis"
Castellana Grotte (BA)
tel. + 39 - 080 4994650 / 651

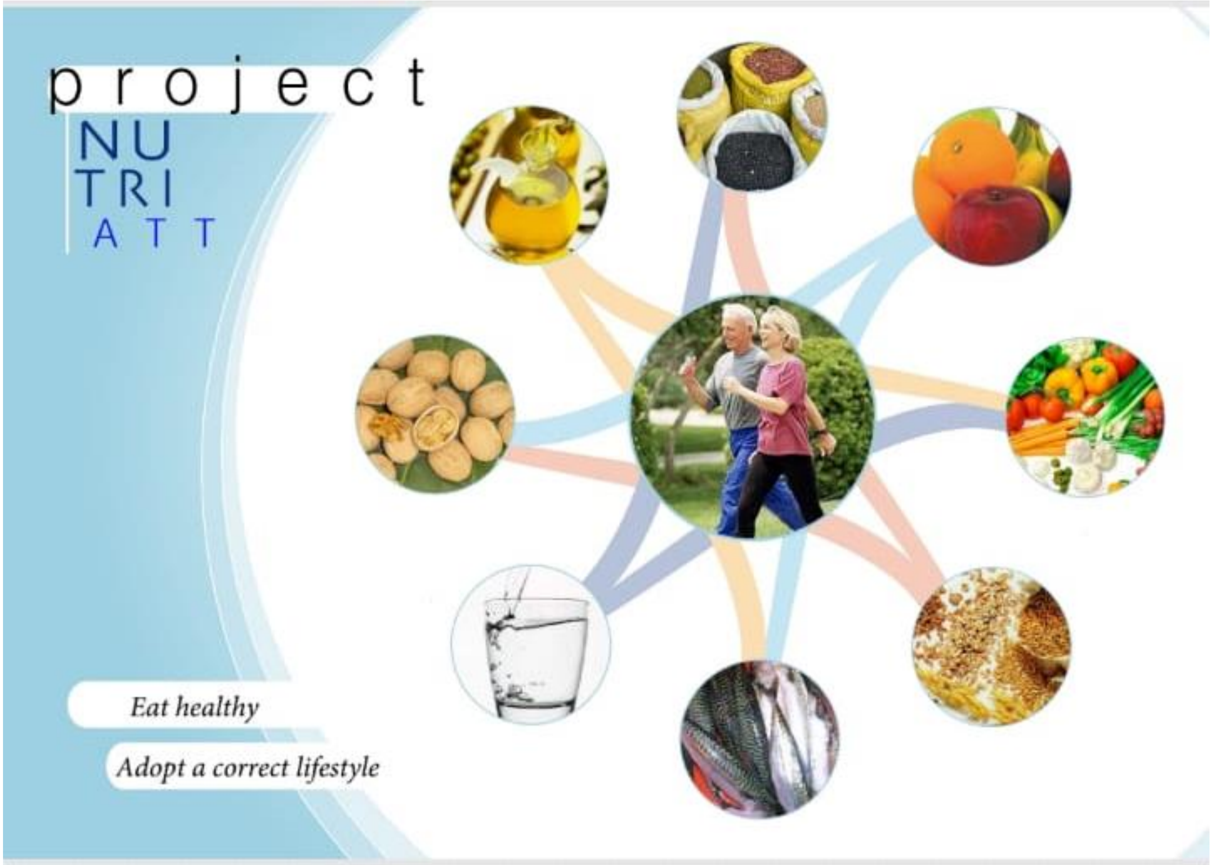

Introduction

Your health is in your hands; you build it each day when you go to the supermarket and choose one food rather than another one, when you cook a lunch or a dinner. In these moments you do dietary choices which influence your health. A wrong diet can determine the appearance of risk factors for cardiovascular diseases and cancer. One of the steps of these diseases is the Non-alcoholic Fatty Liver Disease (NAFLD) (fat liver).

We found that your clinical, laboratory and instrumental data , which came from the NutriEP study you participate in, evidenced that you have NAFLD and then you are at a greater risk to develop cardiovascular disease or tumors (the principal causes of death in Western World).

The Laboratory of Epidemiology and Biostatistics of the IRCCS Saverio de Bellis from Castellana Grotte (Bari) has started up the research project "NutriEpa: EFFECT OF A LOW GLYCEMIC INDEX MEDITERRANEAN DIET ON NON ALCOHOLIC FATTY LIVER DISEASE. A RANDOMIZED CONTROLLED CLINICAL TRIAL" to evaluate the efficacy of the best diet to treat NAFLD".

So, we invite you to participate in this project which will help us to understand the efficacy of the diet to treat NAFLD. Dietary advices described in the following pages are those recommended by the World Health Organization (WHO) and we ask you to follow them. Furthermore we ask you to record your daily diet in this dietary record as long as the duration of the study. This will permit us to measure adherence level to the diet we gave you.

WATCH THE SEMAPHORE!

CHECK YOUR DIETARY CHOICES

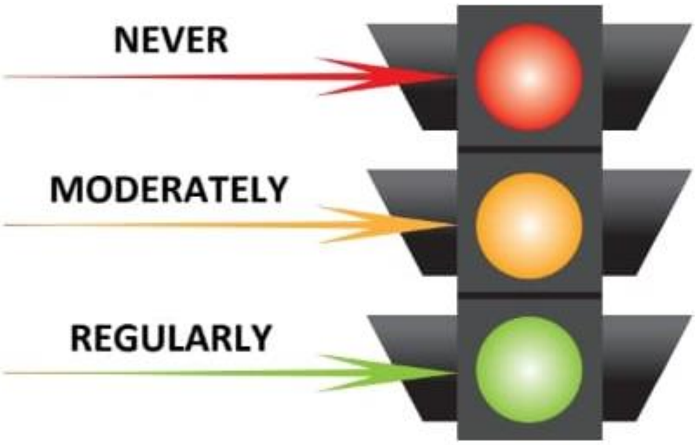

Foods choice and the frequency of their consumption is fundamental to set a correct dietary programm, which day after day should be followed by everyone to keep a satisfying state of health. To direct foods choice towards those which best represent the indications of the **World Health Organization (WHO)** we marked with **GREEN** colour the foods to consume regularly, with **YELLOW** those to consume moderately and with **RED** those to avoid completely.

REGULARLY
1- Raw vegetables (lettuce, tomatoes, cucumbers, celery, carrots, radishes, etc)
2- Steamed or boiled vegetables (beets, turnips, chicory, cauliflower, broccoli, etc.)
45- Dried pulses (lentils, chickpeas, beans, broad beans, soybeans)
46- Own fresh vegetables (peas, beans, green beans)
10- Pasta<br>16- Rice
23- Potatoes
27- Tomato sauce
28- Extra virgin olive oil (raw)
20- White bread
53- Crackers, pretzels, bread sticks, bread and breakfast cereals
34- White meat (chicken, turkey, rabbit)
39- Fish
40- Molluscs and crustaceans
44- Canned tuna
3- Fresh fruit (apples, pears, oranges, grapefruit, kiwi, peaches, etc.)
54- Dry biscuits, toast
64- Coffee
52- Natural water

MODERATELY	max
30- Milk and yogurt	2 times/daily
32- Dairy products (cheese, smoked cheese, cottage cheese)	3 times/weekly
31- Cheese (parmigiano, cheese, etc.)	3 times/weekly
33- Eggs	2 times/weekly (1 egg)
35- Red meat (beef, veal, pork, horse)	2 times/weekly
37- Stew	1 time/weekly
38- Sausages (raw or cooked ham, dried beef, bacon, salami, mortadella, etc.)	2 times/weekly
47- Pizza	1 time/weekly
6- Sweet dried fruit (dried figs, dried dates, prunes, raisins)	2 times/weekly
50- Non carbonated soft drinks (orange juice, coca-cola, fruit juice, etc.)	1 time/weekly
48- Beer	1 time/weekly (max 33 cl.)
49- Wine	2 times/daily (a glass)
61- Jams	2 times/weekly
59- Ice-cream and water ice	2 times/weekly
57- Cakes, sweets, pastries	1 time/weekly

NEVER
51- Alcoholic beverages and spirits (brandy, grappa, liqueurs, whiskey)
24- French fries
67- Fry
68- Sausages and bacon
29- Butter, margarine, mayonnaise, cream
69- High-fat cheeses (mascarpone, gorgonzola, cream cheese)
9- Fruit in syrup and candied sugar
58- Croissants, pastries and snacks
60- Candies and chocolates

Your Dietary Record

Instructions

- It's fundamental to complete the Dietary Record each day immediately after the meal or as soon as possible.
- The Dietry Record is composed of a series of pages, one for each week. In each weekly page, days from Monday to Sunday are divided in breakfast, lunch, dinner and snack.
- Record all the foods you eat and what you drink during the day (Sunday and holydays included).
- Registration consists in taking note, in correspondence to the meal, the identification number of the food/drink or of the food group consumed belonging to the list we enclosed. If the food you ate isn't included in the list, just write its name in the place reserved to the corresponding meal.

Example:

Here below it is shown an hypothetical day of Mr Rossi's Dietary Record.

After each meal Mr Rossi registered what he ate and drank, so he wrote in the box of the specific meal the correponding number of the food he consumed (by checking the foods list).

At breakfast he drank a cup of milk and coffee with a teaspoon of sugar and he ate an apple.

	BREAKFAST	Snack	LUNCH	Snack	DINNER	Snack	PHYSICAL ACTIVITY
MONDAY	30 + 64 + 63<br>3						☐ None<br>☐ Walk<br>☐ Housework<br>☐ Training<br>☐ Other (specify what)

At lunch he had a plate of lentils (without pasta) seasoned with raw extra-virgin olive oil, a cod, a plate of raw unseasoned vegetables and he drank two glasses of wine and a glass of natural water.

	BREAKFAST	Snack	LUNCH	Snack	DINNER	Snack	PHYSICAL ACTIVITY
MONDAY			45 + 28<br>39, 1<br>49 + 49<br>52				☐ None<br>☐ Walk<br>☐ Housework<br>☐ Training<br>☐ Other (specify what)

In the afternoon he had an apple and drank a coffee with a teaspoon of sugar.

	BREAKFAST	Snack	LUNCH	Snack	DINNER	Snack	PHYSICAL ACTIVITY
MONDAY				3<br>64 +63			☐ None<br>☐ Walk<br>☐ Housework<br>☐ Training<br>☐ Other (specify what)

At dinner he had a plate of minestrone soup (without pasta) seasoned with raw extra-virgin olive oil, a slice of whole bread, a pear and drank two glasses of natural water.

	BREAKFAST	Snack	LUNCH	Snack	DINNER	Snack	PHYSICAL ACTIVITY
MONDAY	30+64+63<br>3		45 + 28<br>39, 1<br>49 + 49<br>52	3<br>64 +63	Minestrone soup+28<br>Whole bread<br>3<br>52 + 52		☐ None<br>☒ Walk<br>☐ Housework<br>☐ Training<br>☐ Other (specify what)

In the last column, referred to the physical activity carried out during the day, he put a cross in correspondence to the entry "walk".

SURNAME ______________________ LAST NAME ______________________ CODE ______________

MONTH ______________________ WEEK from ______________________ to ______________

	BREAKFAST	Snack	LUNCH	Snack	DINNER	Snack	PHYSICAL ACTIVITY
Monday							☐ None<br>☐ Walk<br>☐ Housework<br>☐ Training<br>☐ Other (specify what) ______
Tuesday							☐ None<br>☐ Walk<br>☐ Housework<br>☐ Training<br>☐ Other (specify what) ______
Wednesday							☐ None<br>☐ Walk<br>☐ Housework<br>☐ Training<br>☐ Other (specify what) ______
Thursday							☐ None<br>☐ Walk<br>☐ Housework<br>☐ Training<br>☐ Other (specify what) ______
Friday							☐ None<br>☐ Walk<br>☐ Housework<br>☐ Training<br>☐ Other (specify what) ______
Saturday							☐ None<br>☐ Walk<br>☐ Housework<br>☐ Training<br>☐ Other (specify what) ______
Sunday							☐ None<br>☐ Walk<br>☐ Housework<br>☐ Training<br>☐ Other (specify what) ______

Ministry of Health

DE BELLIS

Research Laboratories
I.R.C.C.S. "Saverio De Bellis"
Castellana Grotte (BA)
tel. + 39 - 080 4994650 / 651
